# Internet-Based Treatment for Adults with Depressive Symptoms: Randomized Controlled Trial

**DOI:** 10.2196/jmir.1094

**Published:** 2008-11-20

**Authors:** Lisanne Warmerdam, Annemieke van Straten, Jos Twisk, Heleen Riper, Pim Cuijpers

**Affiliations:** ^3^Trimbos InstituteNetherlands Institute of Mental Health and AddictionUtrechtThe Netherlands; ^2^Department of Clinical Epidemiology and Applied BiostatisticsVU UniversityAmsterdamThe Netherlands; ^1^Department of Clinical PsychologyInstitute for Research in Extramural MedicineVU UniversityAmsterdamThe Netherlands

**Keywords:** Internet, depression, self-help, cognitive therapy, problem solving, randomized trial

## Abstract

**Background:**

Many depressed people do not receive help for their symptoms, and there are various barriers that impede help-seeking. The Internet may offer interesting alternatives for reaching and helping people with depression. Depression can be treated effectively with Internet-based cognitive behavioral therapy (CBT), but a short intervention based on problem solving therapy (PST) could constitute a worthwhile alternative to CBT.

**Objective:**

In this study we evaluated the effectiveness of Internet-based CBT and Internet-based PST in comparison to a waiting list control group (WL), and we determined the differences between the two treatments.

**Methods:**

We conducted a 3-arm randomized controlled trial to compare CBT, PST, and WL. The main inclusion criterion was presence of depressive symptoms (≥ 16 on the Center for Epidemiological Studies Depression scale). CBT and PST consisted of eight and five weekly lessons respectively. Participants were supported by email. Self-report measures of depression, anxiety, and quality of life were completed at pretest and after 5, 8, and 12 weeks.

**Results:**

A total of 263 participants were randomized to the three conditions (CBT: n=88; PST: n=88; WL: n=87). Of the 263 participants, 184 (70%) completed questionnaires after 5 weeks, 173 (66%) after 8 weeks, and 151 (57%) after 12 weeks. Between-group effect sizes for depressive symptoms were 0.54 for CBT after 8 weeks (95% confidence interval (CI): 0.25 - 0.84) and 0.47 for PST after 5 weeks (95% CI: 0.17 - 0.77). These effects were further improved at 12 weeks (CBT: 0.69, 95% CI: 0.41 - 0.98; PST: 0.65, 95% CI: 0.36 - 0.95). For anxiety, effect sizes were also at a medium level. Effect sizes for quality of life were low. The number of participants showing clinically significant change at 12 weeks was significantly higher for CBT (n = 34, 38.6%) and PST (n = 30, 34.1%), compared to WL (n = 0).

**Conclusions:**

Both Internet-based treatments are effective in reducing depressive symptoms, although the effect of PST is realized more quickly.

**Trial Registration:**

International Standard Randomized Controlled Trial Number (ISRCTN): 16823487; http://www.controlled-trials.com/ISRCTN16823487/16823487  (Archived by WebCite at http://www.webcitation.org/5cQsOj7xf).

## Introduction

Depression is known to be one of the most prevalent mental disorders in the world [1] and is expected to be the disorder with the highest disease burden in high-income countries by 2030 [2]. Several trials have shown that there are effective self-help treatments for depression, including Internet-based self-help [3,4]. Still, many depressed people do not seek treatment [5]. Barriers to receiving adequate treatment include a shortage of skilled therapists, costs, and long waiting lists. More personal barriers to talking to a professional therapist include the idea that “talking” does not help, lack of willingness to talk to a stranger about personal problems, and fear of stigma [6].Thus, a major challenge lies in increasing the applicability and accessibility of Internet-based psychological treatments for a broad population with clinically relevant depressive symptoms and simultaneously minimizing contact with a professional therapist.

Most self-help therapies are based on cognitive behavioral therapy (CBT) because of its effectiveness with depression [4,7] and its structured format which makes it very suitable for self-help purposes. It is unknown whether other self-help formats are also effective. Problem-solving therapy (PST) is effective in reducing depression and several other mental health problems [8,9]. As far as we know, there is no study which evaluates Internet-based PST for depression. Recently, a new, generic, PST-based intervention for multiple mental health problems that could be applied through the Internet was developed [10]. As a general framework for the intervention, the model of Bowman and colleagues [11], which is called self-examination therapy, was used. The general idea of self-examination therapy is that subjects learn to regain control over their problems and lives by (1) determining what really matters to them, (2) investing energy only in those problems that are related to what matters, (3) thinking less negatively about the problems that are unrelated, and (4) accepting those situations that cannot be changed. Self-examination therapy was exclusively designed to be a self-administered treatment and has been found to be effective in several studies in the United States [11-14]. In these studies, self-examination therapy was offered in book format, and it is not known whether it also works when given via the Internet.

Our PST-intervention is a Dutch adaptation of self-examination therapy. After adjusting PST for the Internet, the effectiveness of this intervention was shown in patients with different mental health symptoms [10]. A characteristic of this intervention is its short duration of only 5 weeks. It would be interesting to know whether this short, Internet-based intervention works equally well as an 8-week Internet-based CBT intervention, thereby possibly making it a worthwhile alternative.

The current study evaluated two Internet-based interventions with support for adults with elevated depressive symptoms. The goal of this study is twofold. First, we wanted to evaluate the effectiveness of Internet-based CBT and Internet-based PST compared to a waiting list control group. Second, we wanted to determine the differences between the two treatments regarding their effectiveness.

## Method

### Design

This study is a randomized controlled trial with three groups: two Internet-based self-help interventions (CBT and PST) and a waiting list control group (WL). The study was designed to compare the efficacy of each of the two interventions with the WL. The sample size was based on the expected difference in the primary outcome variable (ie, depressive symptoms, between one of the intervention groups and the waiting list control group at post-test). Based on a power of 0.80 in a one-tailed test, an alpha of 0.05, we needed 100 subjects in each condition to show an effect size of 0.40. Therefore, the total sample size was determined at 300.

### Participants

Participants were recruited through advertisements in daily and weekly newspapers and through banners on general websites such as Google and on websites relating to mental health problems. Recruitment took place during two periods, in August/September 2006 and in January/February 2007. Application took place via a website. After application, subjects received a brochure about this study and an informed consent form by post. After giving informed consent, participants received the baseline questionnaire by email. The study protocol was approved by the Medical Ethics Committee of the VU University Medical Center.

All adults aged 18 years and older with depressive symptoms who were willing to participate in a self-help course, were eligible for this study. The main inclusion criterion was a score of 16 or more on the Center of Epidemiologic Studies Depression—scale (CES-D) [15]. Participants with more severe symptoms of depression (indicated by a CES-D score of 32 or higher) were advised to consult their general practitioner but could participate in the study. Other inclusion criteria were: sufficient knowledge of the Dutch language, access to Internet, and having an email address. No exclusion criteria were defined for this study.

### Randomization

Randomization took place at an individual level after the baseline measurement and one week before the start of the interventions. Received baseline questionnaires were numbered in order of arrival. Subjects were randomized into three groups, two intervention groups and a waiting list control group. We used block randomization, with each block containing 9 allocations. An independent researcher made the allocation schedule with a computerized random number generator. Immediately after randomization, subjects were informed about the randomization outcome by email.

### Interventions

#### Problem Solving Therapy (PST)

Our PST-based intervention is a Dutch adaptation of SET from Bowman [11]. We added more information, examples, exercises, and forms. PST consisted of three steps. First, the subjects described what really matters to them. Second, they wrote down their current worries and problems. They divided these problems into three categories: (a) unimportant problems (problems unrelated to the things that matter to them), (b) solvable problems, and (c) problems which cannot be solved (eg, the loss of a loved one). For each of these three types of problems a different strategy is proposed to solve the problems or to learn to cope with the unimportant and unsolvable ones. The core element of PST is to address the solvable problems by the following six-step procedure: describing the problem, brain-storming, choosing the best solution, making a plan for carrying out the solution, actually carrying out the solution, and evaluation. During the third and last step, the subjects made a plan for the future in which they described how they would try to accomplish those things that matter most to them. The course took 5 weeks and consisted of one lesson a week. The intervention made use of information, exercises, examples of people applying the principles of PST, and a built-in feedback system. There were no audio-visual aids. See Multimedia Appendix 1 for a screenshot of the PST intervention.

#### Cognitive Behavioral Therapy (CBT)

The CBT intervention was developed by the Trimbos Institute—The Netherlands Institute of Mental Health and Addiction. This intervention is based on the “Coping with Depression” course (CWD) [16], Dutch version [17].CWD is a highly structured psycho-educational form of cognitive behavior therapy for depression. Theoretically, this course is based on the social learning theory according to which depression is associated with a decrease in pleasant and an increase in unpleasant person-environment interactions. People’s problems are viewed as behavioral and cognitive patterns which can be unlearned or relearned.

Like CWD, CBT in this study included psycho-education and focused on skills such as relaxation, cognitive restructuring (including worrying), social skills, and how to increase the number of pleasant events. CBT consisted of eight lessons, one lesson a week. The ninth lesson took place 12 weeks later. See Multimedia Appendix 1 for a screenshot of the CBT intervention. The intervention made use of information, exercises, and audio-visual aids with instructions during the lessons and examples of people applying the principles of CBT. This Web-based intervention has been found to be effective in older adults with sub-threshold depression, both in the short-term [18] and at one-year follow-up [19] and was found to be superior to a group intervention.

#### Support

Subjects in both intervention groups received support during the intervention period by email from Master-level students of clinical psychology. Students underwent training of 6 hours in total. This training was given by the first author of this article. Support was directed at helping the participant to work through the intervention, and not at developing a therapeutic relationship or giving direct or individual advice on how to cope with depressive symptoms or other problems. The content of the feedback consisted of three aspects: showing empathy by letting participants know that the coach had read the assignments, being positive by giving compliments on what the participant had done, and giving suggestions on how to continue with the course.

Every week, a standardized email was sent to the participants. This email communicated the lesson of that week and the date on which the assignments were to be sent to their coach. Participants received feedback within three working days. All feedback was checked by the first author and if necessary comments were added before it was sent to the participants. The average time spent on each participant by a therapist to provide feedback and answer questions via email is estimated to be 20 minutes per week, resulting in approximately 100 minutes for PST and 160 minutes for CBT.

### Outcome Measures

All participants were contacted for outcome assessments at 5, 8, and 12 weeks after the start of the interventions. All questionnaires were administered on the Internet. Participants received an email with a link to the questionnaire.

#### Depressive Symptoms

The Center for Epidemiological Studies Depression scale (CES-D, Dutch version) [15] was the primary outcome measure for depressive symptoms. The CES-D is widely used for identifying people with depressive symptomatology. Scores of 16 and higher represent a clinically significant level of depressive symptoms. The validity of the CES-D has been tested in different populations [20-22]. The CES-D consists of 20 items and the total score varies between 0 and 60 with higher scores indicating more depression [15].

#### Anxiety

The anxiety subscale of the Hospital Anxiety and Depression Scale (HADS) was used for the measurement of anxiety symptoms [23]. The anxiety subscale consists of 7 items. Scores range from 0 to 21 with higher scores indicating more anxiety. The HADS showed good homogeneity and reliability, with Cronbach’s alpha ranging from .81 to .84 in various normal and clinical Dutch samples [23].

#### Quality of Life

Quality of life was assessed with the EuroQol Questionnaire (EQ5D) [24], which is a validated tool for measuring general health-related quality of life. It consists of 5 items (mobility, self-care, usual activities, pain/discomfort, and anxiety/depression), each of which is rated as causing “no problems”, “some problems”, or “extreme problems”. The EQ5D thus distinguishes 486 unique health states. Each unique health state has a utility score which ranges from 0 (poor health) to 1 (perfect health). We used this single EQ5D summary index score.

### Statistical Analyses

#### Missing Values

All analyses were performed on the intention-to-treat sample. The Linear Mixed Modeling (LMM) procedure was used for all analyses to estimate missing values. LMM includes incomplete cases in the analysis and employs restricted maximum likelihood estimation to calculate parameter estimates. LMM assumes that missing data are missing at random.

#### Baseline Differences and Attrition

Baseline differences in demographic and clinical characteristics were investigated using Chi-square tests, *t*-tests, and analysis of variance (ANOVA). Attrition was defined as completing none or one of the three post-treatment measures.

#### Treatment Differences

LMM was used to investigate treatment differences. As we were interested in the differences between groups at each time period, we treated time as a categorical variable. Treatment condition was treated as a fixed effect. The intercept was included as a random effect.

#### Effect Sizes

Between-group effect sizes were calculated according to Cohen’s d. Effect sizes of 0.8 can be assumed to be large, while effect sizes of 0.5 are moderate, and effect sizes of 0.2 are small [25]. Estimated data from the LMM procedure were used to calculate effect sizes.

#### Clinically Significant Change

Clinical significant change was determined with norms for the outcome measure and with the Reliable Change Index [15,26]. We used the cut-off score of 16 on the CES-D as an indication of recovery. RC was used as an index for improvement. Results were analyzed for the intention-to-treat sample as they were for the sample who completed questionnaires.

#### Completion Status

LMM was used to investigate differences in development of depressive symptoms between treatment completers, non-completers, and WL. Time was treated as a continuous covariate. Completers were defined as subjects who completed all lessons.

## Results

### Participants

**Figure 1:**
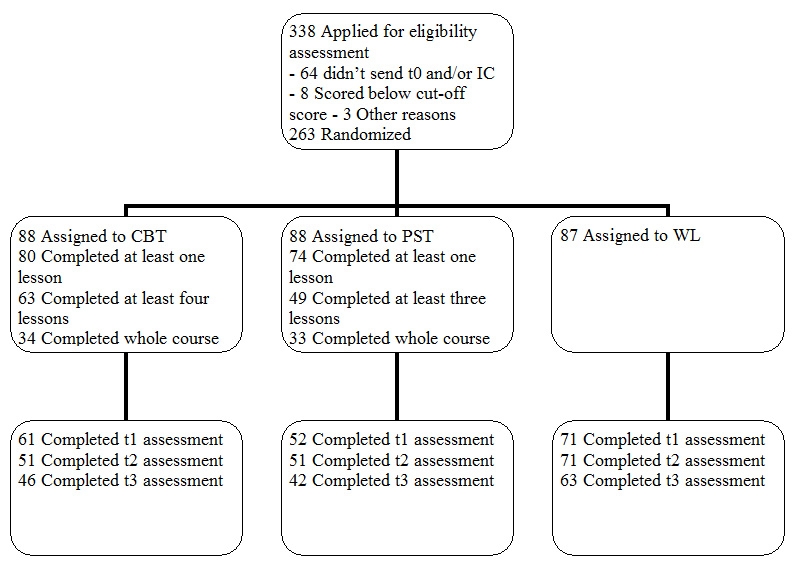
Participant flow

Figure 1 shows the progress of participants through the trial. Of the 338 individuals who were potentially interested in participating, 64 did not send back the baseline questionnaire or did not give informed consent. From 274 subjects we received baseline questionnaires and written informed consent. Of these, 8 did not score above the cut-off of 16 on the CES-D and 3 subjects decided not to participate for other reasons. The remaining 263 participants were randomized to one of the three conditions. Table 1 presents demographic characteristics. Participants were mainly female (71%, n = 187). The mean age was 45 years (SD: 12.1). Almost all subjects came from the Netherlands (92%, n = 243). A majority of the participants (64%, n = 168) had completed higher vocational education or university. The mean score of the 263 participants on the CES-D at baseline was 31.7 (SD: 7.5, median: 31.0). There were no statistically significant differences between the groups at baseline with respect to demographics or symptoms (Table 1 and Table 2).

### Attrition

Attrition rates for the full sample were 30% (n = 79) at the 5-week assessment, 34% (n = 90) at 8 weeks, and 43% at 12 weeks (n = 112). Reasons for the high level of attrition were unknown. Some participants dropped out because of, for example, other treatment, feeling better, lack of time, and problems understanding the computer program, but the majority did not specify any reason. There were significant differences in attrition rates between the three conditions. Attrition rates were lower in the control group than in both intervention groups at all assessments (5wk WL: 18%, n = 16, 5wk  CBT: 31%, n = 27, 5wk PST: 41%, n = 36, χ^2^_2,263_ = 10.58, *P* = .01; 8wk WL: 18%, n = 16, 8wk CBT: 42%, n = 37, 8wk PST: 42%, n=37, χ^2^_2,263_ = 14.47, *P* = .001; 12wk WL: 28%, n = 24, 12wk CBT: 48%, n = 42, 12wk PST: 52%, n = 46, χ^2^_2,263_ = 12.33, *P* = .002).

Some statistically significant differences at baseline were detected between participants who completed post-treatment measures and those who did not. Participants who completed post-treatment measures were more likely to have been born in the Netherlands (95%, n = 161) than participants who didn’t (87%, n = 82, χ^2^_1,263_ = 5.55, *P* = .02), and they were also older (46.6 and 41.9 years respectively, *t*_259_ = -2.91, *P* = .004).

**Table 1:** Demographic characteristics at baseline

	All (n = 263)	CBT (n = 88)	PST (n = 88)	WL (n = 87)	Statistic
**Age (years)**	45.0	45.7	45.1	44.1	F_2,258_ = 0.40, *P*= .67
**Female**	187 (71.1)	61 (69.3)	57 (64.8)	69 (79.3)	χ^2^_2_ = 4.71, *P*= .10
**Country of birth**					χ^2^_2_ = .12, *P*= .94
The Netherlands	243 (92.4)	81 (92.0)	82 (93.2)	80 (92.0)	
**Education^a^**					χ^2^_4_ = 5.96, *P*= .20
lower	23 (8.7)	9 (10.2)	5 (5.7)	9 (10.3)	
middle	72 (27.4)	26 (29.5)	18 (20.5)	28 (32.2)	
higher	168 (63.9)	53 (60.2)	65 (73.9)	50 (57.5)	
**Paid job**	135 (53.8)	43 (52.4)	43 (50.6)	49 (58.3)	χ^2^_2_ = 1.12, *P*= .58
Note: Data are presented as n (%) of participants unless otherwise indicated.					

^a^lower = primary education or lower general secondary education, middle = intermediate vocational education or high school, high = higher vocational education or university

### Effects of the Interventions

**Figure 2:**
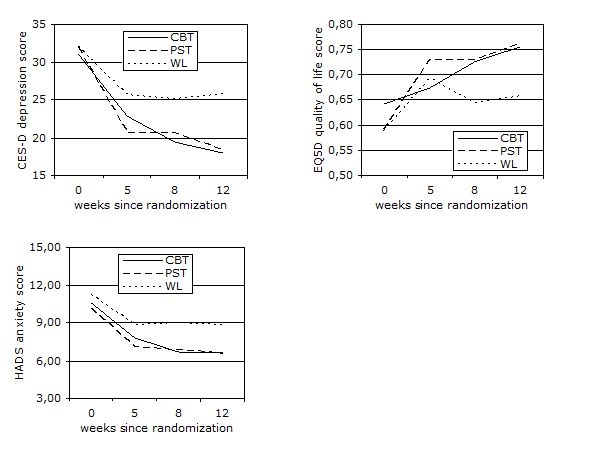
Estimated trajectories of improvement in depression, anxiety and quality of life scores by treatment assignment

Table 2 reports the estimated means and standard deviations as produced by the linear mixed model procedure, using the intention-to-treat sample. These means are used to produce the estimated trajectories in Figure 2. There was significant overall improvement over time for all groups on the CES-D, F_3,543_ = 124.57, *P* < .001. In addition, results revealed significant group x time interaction effects on the CES-D, F_6,543_ = 5.61, *P* < .001. As shown in Figure 2, mean depression scores after 5 weeks were significantly lower in PST than in WL, *t*_592_ = -3.01, *P* = .002. After 8 weeks, both CBT and PST showed significantly lower depression scores than WL (CBT: *t*_598_ = -3.64, *P* < .001, PST: *t*_596_ = -2.89, *P* = .004). Also after 12 weeks, CBT and PST showed significantly lower depression scores than WL (CBT: *t*_635_ = -4.73, *P* < .001, PST: *t*_650_ = -4.34, *P* < .001). No differences were found in depression scores between CBT and PST at each assessment.

Regarding anxiety scores, significant overall improvement over time was found for all groups on the HADS, F_3,538_ = 81.74, *P* < .001 (Figure 2). After 5 weeks, PST showed significantly lower mean anxiety scores than WL, *t*_582_ = -2.78, *P* = .006. After 8 weeks, both CBT and PST showed significantly lower anxiety scores than WL (CBT: *t*_588_ = -3.63, *P* < .001, PST: *t*_586_ = -3.34, *P* = .001). Also after 12 weeks, CBT and PST showed significantly lower anxiety scores than WL (CBT: *t*_627_ = -3.51, *P* < .001, PST: *t*_642_ = -3.35, *P* = .001). No differences were found in anxiety scores between CBT and PST at each assessment.

As shown in Figure 2, there was significant overall improvement over time for all groups on the EQ5D, F_3,502_ = 23.25, *P* < .001. Furthermore, results showed significant group x time interaction effects on the EQ5D, F_6,501_ = 2.97, *P* = .007. No differences were found between each of the treatments and WL after 5 weeks. After 8 weeks, both CBT and PST showed significantly higher quality of life scores than WL (CBT: *t*_560_ = 2.11, *P* = .04, PST: *t*_564_ = 2.20, *P* = .03). After 12 weeks, CBT and PST indicated significantly higher quality of life scores than WL as well (CBT: *t*_588_ = 2.41, *P* = .02, PST: *t*_613_ = 2.52, *P* = .01). No differences were found in quality of life scores between CBT and PST at each assessment.

All effect sizes are presented in Table 3. Effect sizes were based on the intention-to-treat sample, using the estimated data from Table 2. The between-group effect sizes were around a medium level for depression and anxiety. Low effect sizes were found for quality of life. The highest values were found for depression after a 12-week follow-up: CBT: 0.69 (95% CI: 0.41 - 0.98), PST: 0.65 (95% CI: 0.36 - 0.95). The lowest value was found for PST on quality of life scores, d = 0.14, which was non-significant.

**Table 2:** Estimated outcomes of CBT and PST on depression, anxiety, and quality of life

Measure and treatment condition	Baseline M (SD)	5 weeks M (SD)	8 weeks M (SD)	12 weeks M (SD)
**CES-D**				
CBT	31.2 (9.3)	22.9 (10.6)	19.4 (11.3)	17.9 (11.7)
PST	31.9 (9.3)	20.6 (11.2)	20.6 (11.3)	18.4 (12.1)
WL	32.1 (9.3)	25.6 (9.9)	25.2 (9.9)	25.8 (10.4)
**HADS**				
CBT	10.6 (3.6)	7.8 (4.1)	6.7 (4.4)	6.6 (4.5)
PST	10.2 (3.6)	7.1 (4.3)	6.9 (4.4)	6.6 (4.7)
WL	11.3 (3.6)	8.9 (3.9)	9.0 (3.8)	8.9 (4.0)
**EQ5D**				
CBT	0.64 (0.18)	0.68 (0.27)	0.73 (0.27)	0.76 (0.27)
PST	0.59 (0.18)	0.73 (0.27)	0.73 (0.27)	0.76 (0.27)
WL	0.59 (0.18)	0.69 (0.27)	0.65 (0.27)	0.66 (0.27)

**Table 3:** Effect sizes (95% CI)

Measure and treatment condition		5 weeks	8 weeks	12 weeks
CES-D	CBT		0.54 (0.25 - 0.84)	0.69 (0.41 - 0.98)
	PST	0.47 (0.17 - 0.77)		0.65 (0.36 - 0.95)
HADS	CBT		0.54 (0.25 - 0.83)	0.52 (0.23 - 0.81)
	PST	0.42 (0.12 - 0.72)		0.50 (0.21 - 0.80)
EQ5D	CBT		0.30 (0.02 - 0.59)	0.36 (0.07 - 0.65)
	PST	0.14 (-0.14 - 0.42)		0.38 (0.09 - 0.68)

### Clinically Significant Change

Data on clinically significant change are presented in Table 4. Rates are reported for participants who were randomly assigned to the conditions (estimated) as well as for participants who completed questionnaires (observed).

Estimated results showed significant between-group differences in terms of clinically significant change on the CES-D. Improvement and recovery after 5 weeks more often occurred in PST (n = 18) than in CBT and in WL, χ^2^_2,263_ = 38.43, *P* < .001. After 8 and 12 weeks, both CBT and PST showed more improvement and recovery than WL (8 weeks: χ^2^_2,263_ = 28.73, *P* < .001, 12 weeks: χ^2^_2,263_ = 42.31, *P* < .001). The number of participants showing clinically significant change at 12 weeks was n = 34 for CBT, n = 30 for PST, and n = 0 for WL. Observed results also showed significant between-group differences at each assessment (5 weeks: χ^2^_2,184_ = 9.63, *P* = .008, 8 weeks: χ^2^_2,173_ = 7.0, *P* = .03, 12 weeks: χ^2^_2,151_ = 11.57, *P* = .003).

**Table 4:** Proportions of participants reaching the criteria of clinically significant change on the CES-D as defined by Jacobson and Truax (1991)

	5 weeks, No. (%)		8 weeks, No. (%)		12 weeks, No. (%)	
Treatment Condition	Estimated	Observed	Estimated	Observed	Estimated	Observed
CBT	0 (0.0)	11 (18.0)	26 (29.5)	21 (41.2)	34 (38.6)	18 (39.1)
PST	18 (20.5)	19 (36.5)	18 (20.5)	20 (39.2)	30 (34.1)	17 (40.5)
WL	0 (0.0)	10 (14.1)	0 (0.0)	15 (21.1)	0 (0.0)	9 (14.3)

### Treatment Completers Versus Non-completers

Many participants failed to complete the whole course. Of those participants assigned to CBT and PST, 8 (9.1%) versus 14 (15.9%) completed no lesson at all. Of those assigned to CBT, 63 (71.6%) participants completed at least four lessons and 34 (38.6%) completed all eight. Of those assigned to PST, 49 (55.7%) participants completed three or more sessions and 33 (37.5%) finished the whole course. More completers had received higher education in contrast to the non-completers (75% vs 60.4%),χ^2^_1,178_ = 4.1, *P* = .04. Regarding clinical characteristics, completers showed significantly lower depression scores at baseline than non-completers (29.8 vs 32.8), *t*_176_ = 2.69, *P* = .008. In addition, quality of life scores were significantly higher among completers (0.68 vs 0.57), *t*_165_ = -3.38, *P* = .001.

We investigated differences in development of depression scores between treatment completers, non-completers, and WL, using the intention-to-treat sample. The interaction between completion status and time was significant F_2,578_ = 12.58, *P* < .001. Both completers and non-completers showed lower depression scores over time than WL (completers: beta = -2.15, *t*_537_ = -4.11, *P* < .001, non-completers: beta = -2.56, *t*_608_ = -4.33, *P* < .001). No differences were found in improvement of depressive symptoms between completers and non-completers (beta = .41, *t*_596_ = 0.68, *P* = .50). Table 5 presents the observed outcomes on depression for participants who completed questionnaires.

**Table 5:** Descriptive statistics of treatment completers, non-completers, and waiting list (WL) on depression

Completion status	N	Baseline M (SD)	N	5 weeks M (SD)	N	8 weeks M (SD)	N	12 weeks M (SD)
completers	72	29.8 (6.8)	67	20.0 (9.2)	65	17.9 (9.2)	62	16.5 (10.5)
non-completers	106	32.8 (7.9)	48	23.4 (10.4)	39	22.5 (10.5)	28	18.9 (9.9)
WL	85	32.0 (7.5)	69	25.1 (9.2)	69	24.9 (11.5)	61	26.2 (10.9)

## Discussion

### Principal Results

The results from the present study show that Internet-based CBT and Internet-based PST are both effective in reducing depressive symptoms in comparison to a waiting list control group. These results were visible directly after treatment and 12 weeks after baseline. There is no indication that one is more effective than the other, although the effects are realized faster by PST than by CBT. Both Internet-based treatments had medium effect sizes for depression after treatment (CBT: d = 0.54, PST: d = 0.47), and these effects were further improved at 12 weeks (CBT: d = 0.72, PST: d = 0.66). Furthermore, 34 participants of CBT (38.6%) and 30 of PST (34.1%) were improved and recovered to a clinically significant degree at follow-up. The secondary outcomes (symptoms of anxiety and quality of life) also showed significant gains over time for both treatment groups, but again, no differences could be demonstrated between them.

### Comparison with Prior Work

Both treatments showed a fast improvement during the first 5 weeks. Rapid improvement at the beginning of treatment is a common finding [27]. Nevertheless, the fast improvement during PST is striking. Perhaps the focus at the beginning of the treatment (activity scheduling for CBT and problem-solving for PST) affects speed of improvement, although both activity scheduling and problem-solving are effective cognitive behavioral strategies [8,28]. More plausible is the role of non-specific factors like expectations. For example, the expectation that symptoms reduce within 5 weeks, could lead to more rapid improvement in PST. It would be worthwhile to shorten the CBT intervention to see if the same effects could be reached as with an 8-week intervention.

The effect sizes we found for depression are somewhat larger than the effect sizes for a subgroup of studies about Internet-based treatment for depression reported in a recent meta-analysis [4]. The interventions in this subgroup of studies had no support, which could be a reason for the difference in effect size. Only one study about depression treatment including support showed a high effect size [29]. With regard to clinical change, the proportion of improved and recovered participants is roughly in line with some other studies [10,18,30]. It is, however, not often that clinically significant change is reported, making it difficult to say which proportions are commonly found.

A crucial problem of self-help is the amount of treatment participants receive. The level of completers in our study (38%) is relatively low in comparison to other trials about Internet-based self-help for depression [29,31,32]. The benefits of these interventions, when taking into consideration the population as a whole, however, could be huge. At a relatively low cost, it’s possible to reach and treat many people with Internet-based therapies, as compared to traditional therapies. It should be noted that we used a strict criterion to define completion. To increase completion rates, telephonic support could be considered in addition to, or instead of, email support [33]. The pace of one lesson per week may have been too rapid, and giving an extra two weeks, for example, could have led to higher completion rates.

Non-completers had lower levels of education and more clinical symptoms at baseline than completers. In fact, our whole sample had a higher education level in comparison to the general population. This raises the question about the suitability of these interventions for people with lower levels of education or more severe symptoms. The importance of completing the whole treatment is unclear. We found that non-completers improved more than those on the waiting list, and no differences were found between participants who completed all lessons and participants who completed fewer lessons.

### Limitations

A number of limitations should be noted. First, we were faced with a high attrition rate, which is a general problem in Internet interventions [34]. Attrition in the control group was significantly lower than that of both treatment groups. We could find no indications for selection bias since we could not demonstrate clear baseline differences between participants who completed questionnaires and participants who did not (except for age and country of birth). The bias that still might have been introduced was accounted for by estimating all missing data (based on restricted maximum likelihood) and performing intention-to-treat analyses. Nevertheless, estimating data and using “imputed” values might have led to unreliable estimates.

It’s also possible that our methods for recruiting people could have led to selection-bias. Therefore, the results may not apply to all depressed people (eg, clinical populations), but we do think that the depression scores of the participants in this study (31.7) are well above the normal range scores and represent clinical forms of depression. A sample of self-referred elders with depressive symptoms and two psychiatric patient samples showed mean CES-D scores of respectively 25.9, 24.4, and 39.1 [21,35].

Another limitation concerns the (lack of) diagnosis. Self-report was used to include participants. However, one of the potential benefits of internet-delivered treatments is that people can stay at home. Requiring participants to come in for a clinical assessment would therefore introduce a limitation. From an economic perspective, the idea of Internet-based self-help without therapist contact is attractive because costs are saved which could be allocated to patients with more extensive care needs.

A methodological issue concerns the comparison of interventions with a different duration. We remedied this obstacle by reporting the effect size, a standardized measure, which makes it possible to compare the effect of CBT at 8 weeks with PST at 5 weeks. In addition, participants were not blind to their condition, which is inherent to studies of psychotherapy in general, and could introduce some bias. Furthermore, our study was limited by a short follow-up period of 12 weeks.

### Future Research and Implications

Future research on Internet-based treatment for depression would benefit from evaluations in other populations. Besides the fact that the effective mechanisms of treatment are still unclear, the cost-effectiveness of Internet-based treatments also needs to be investigated.

Clinical implications of the results in this study are twofold. First of all, this study shows promising results for a short term PST-based intervention. It gives some insight into the necessary length of a treatment to reach a significant reduction of depressive symptoms. Short interventions reduce costs in terms of time and effort, for the depressed participant as well as for the supporting therapists. The second implication concerns the possibility of fitting a short, generic intervention, like the Internet-based PST in this study, within a stepped-care path. The generic nature of PST makes it suitable to address different kinds of symptoms. This is of practical use because of the large co-morbidity of diverse psychological symptoms.

### Conclusions

In summary, the results of this study provide support for the use of a short Internet-based problem solving therapy with depressive symptoms. The results seem to be as good as other, longer, Internet-based therapies.

## Multimedia Appendix 1

Screenshots of the PST and CBT interventions

## Abbreviations

CBT: Internet-based cognitive behavioral therapy
CES-D: Center for Epidemiological Studies Depression scale
CI: confidence interval
CWD: Coping with Depression course
EQ5D: EuroQol questionnaire
HADS: Hospital Anxiety and Depression Scale
LMM: linear mixed modeling
PST: Internet-based problem solving therapy
RC: reliable change index
SET: self-examination therapy
WL: waiting list control group
