# Supplementary material for: Internet-Based Treatment for Adults with Depressive Symptoms: Randomized Controlled Trial
Source: J Med Internet Res. 2008 Nov 20;10(4):e44. doi: 10.2196/jmir.1094 (PMC2629364; doi:10.2196/jmir.1094)

Multimedia Appendix: Screenshots

J Med Internet Res 10(4):e44
http://www.jmir.org/2008/4/e44/

PST Intervention

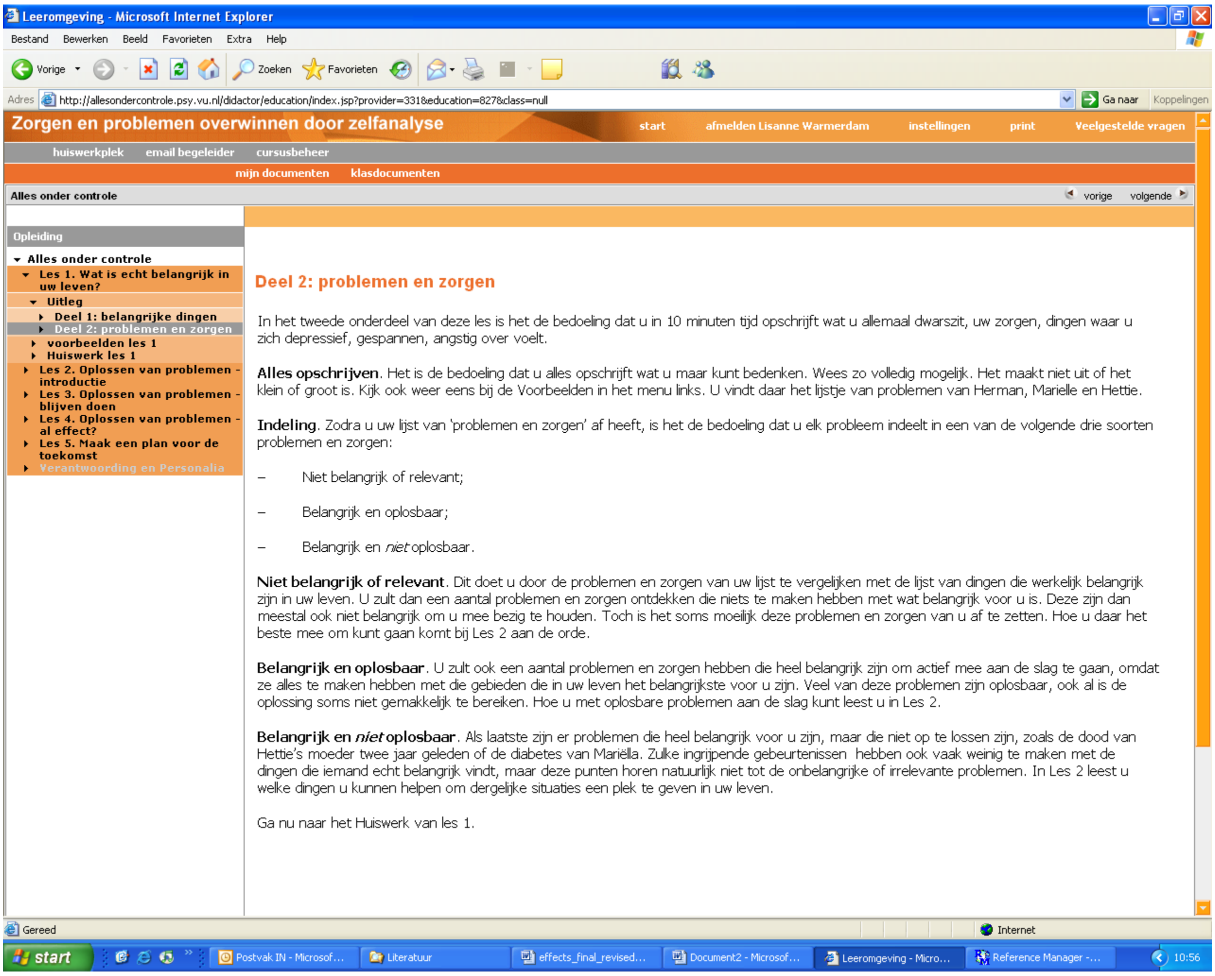

CBT Intervention

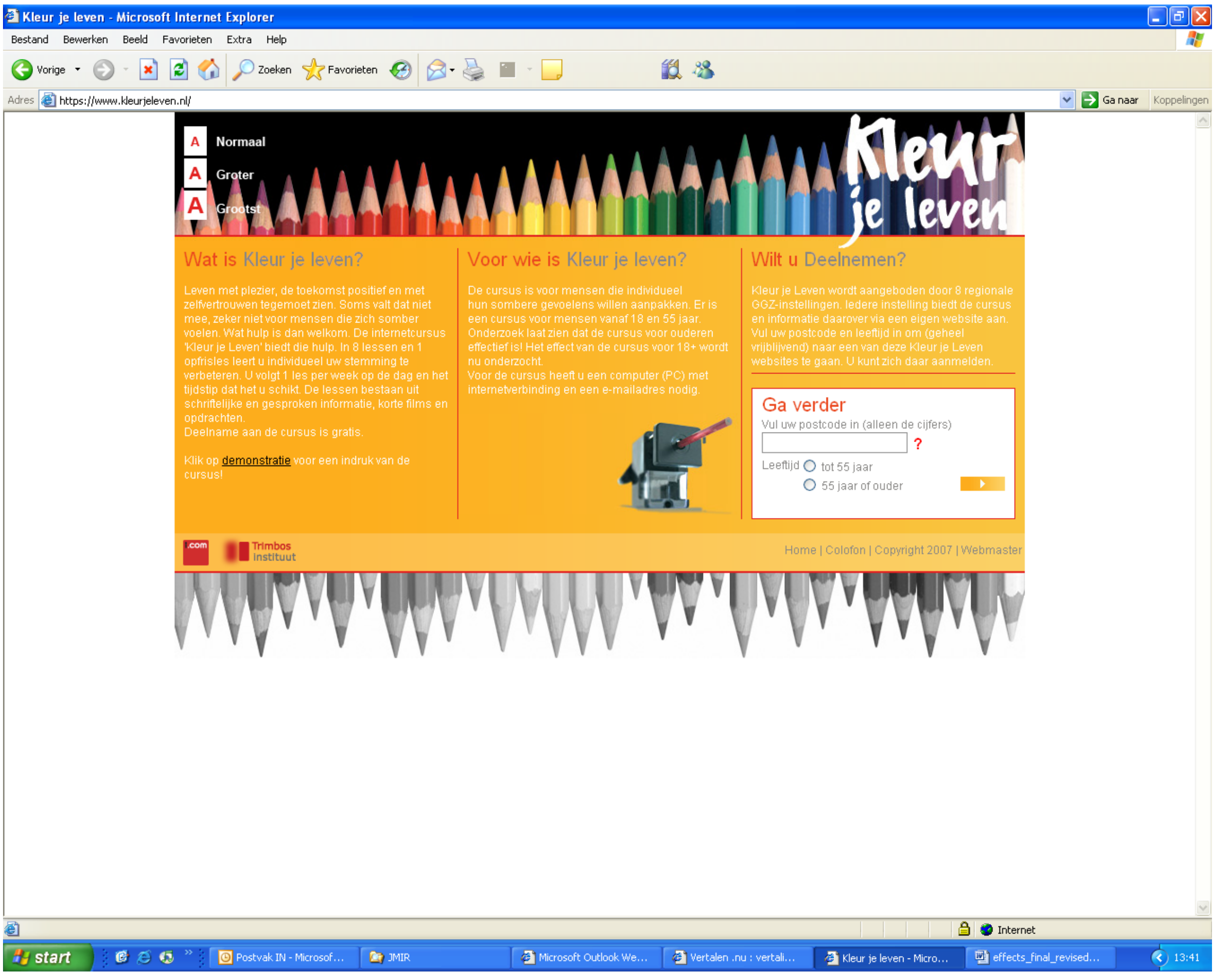

Supplement: Supplementary file 1 [file jmir_v10i4e44_app1.pdf]
